# Evaluation of the Effectiveness of Herbal Components Based on Their Regulatory Signature on Carcinogenic Cancer Cells

**DOI:** 10.3390/cells10113139

**Published:** 2021-11-12

**Authors:** Fazileh Esmaeili, Tahmineh Lohrasebi, Manijeh Mohammadi-Dehcheshmeh, Esmaeil Ebrahimie

**Affiliations:** 1 Department of Plant Bioproducts, National Institute of Genetic Engineering and Biotechnology (NIGEB), Shahrak-e Pajoohesh, km 15, Tehran-Karaj Highway, Tehran P.O. Box 14965/161, Iran; nasibe.esmaili@gmail.com (F.E.); lohrasebi@nigeb.ac.ir (T.L.); 2 School of Animal and Veterinary Sciences, The University of Adelaide, Adelaide, SA 5371, Australia; manijeh.mohammadidehcheshmeh@adelaide.edu.au; 3 Genomics Research Platform, School of Life Sciences, College of Science, Health and Engineering, La Trobe University, Melbourne, VIC 3086, Australia; 4 School of BioSciences, The University of Melbourne, Melbourne, VIC 3010, Australia

**Keywords:** meta-analysis, supervised machine learning, decision tree, transcription factors, herbal compound

## Abstract

Predicting cancer cells’ response to a plant-derived agent is critical for the drug discovery process. Recently transcriptomes advancements have provided an opportunity to identify regulatory signatures to predict drug activity. Here in this study, a combination of meta-analysis and machine learning models have been used to determine regulatory signatures focusing on differentially expressed transcription factors (TFs) of herbal components on cancer cells. In order to increase the size of the dataset, six datasets were combined in a meta-analysis from studies that had evaluated the gene expression in cancer cell lines before and after herbal extract treatments. Then, categorical feature analysis based on the machine learning methods was applied to examine transcription factors in order to find the best signature/pattern capable of discriminating between control and treated groups. It was found that this integrative approach could recognize the combination of TFs as predictive biomarkers. It was observed that the random forest (RF) model produced the best combination rules, including AIP/TFE3/VGLL4/ID1 and AIP/ZNF7/DXO with the highest modulating capacity. As the RF algorithm combines the output of many trees to set up an ultimate model, its predictive rules are more accurate and reproducible than other trees. The discovered regulatory signature suggests an effective procedure to figure out the efficacy of investigational herbal compounds on particular cells in the drug discovery process.

## 1. Introduction

Plants are an important source of novel pharmacologically active compounds with many novel drugs. Approximately 25% of natural-based medicines were directly or indirectly derived from medicinal herbs [1]. For instance, it has been well documented that natural compounds isolated from medicinal plants exhibit considerable anti-cancer activity with low toxicity [2]. Therefore, developing new anti-cancer drugs based on plants is one of the main strategies in the modern drug discovery era [1].

Detecting new and innovative drugs from natural resources remains a complicated, time-consuming, and expensive project [3,4]. Recently, cancer cell line profiling and drug sensitivity research revealed precious information about the therapeutic potential of drugs. Discovering genomic and molecular features from cancer cell lines can help to predict their sensitivity to drugs and provide valuable information about the possible mechanisms of those drugs’ action [5].

Among the genetic factors, TFs play a crucial role in several cellular functions, such as cell growth and development, response to changes in their internal and external environment, and control of cell cycle and carcinogenesis. TFs bind to the promoter region of genes and regulate the transcription of DNA fragments to RNA messenger. Their primary responsibility is governing gene expression in the correct cell at the precise time [6].

Regarding the cancer area, transcriptional dysregulation triggers many disorders leading to tumor progression and drug resistance acquisition [7]. Such dysregulation occurs through direct or indirect mechanisms, including point mutations, chromosomal translocations, alteration of expression, gene amplification or deletion, non-coding DNA mutations, DNA methylations, and histone modifications [8,9,10]. These genomic alterations cause perturbations in gene expression, particularly silencing tumor-suppressive TFs and activating oncogenic TFs [7,11]. For instance, function loss of P53, a tumor suppressor TF, was observed in about 50% of cancers [12]. On the contrary, activating oncogenic TFs such as NF-kB, STAT3, and AP1 regulates tumor initiation and progression [13]. Additionally, recent studies have indicated that some of TFs can possess both tumor-suppressor and oncogenic roles depending on the type of cancer [7]. Consequently, TFs play an important role in cancers through modulating multiple downstream signaling pathways, and they can be proposed as prominent predictive biomarkers candidates.

Effective computational methods and a substantial number of samples are required to identify predictive biomarkers. Such biomarkers help to evaluate the drug effectiveness, which in turn helps reduce time and money spent on curing diseases [14,15]. Advanced methodologies in high-throughput transcriptomic data have generated large-scale public datasets, which characterize cell response to a drug. These datasets offered an opportunity to clarify diseases’ molecular mechanisms and presented significant awareness of the drug. For example, the RNA-seq technique, as a powerful tool for genome-wide transcriptional profiling, can offer comprehensive information on the cellular status and how this status alters following various treatments or conditions [16].

However, exploring biomarker genes using individual transcriptomic studies is challenging due to low replications, low data repeatability, and significant prediction error. Meta-analysis is a way to deal with these deficiencies by gathering as much relevant data as possible from a range of available experiments [17]. Merging individual research outcomes with almost similar objectives can improve the results’ generalizability and statistical capability [18].

Additionally, the availability of transcriptomic datasets in gene expression databases rendered a significant opportunity to apply machine learning models to predict drug activity. Recently, supervised machine learning models are frequently being employed on the omics data to identify ‘druggable’ genetic targets and drug response-predictive biomarkers [14,19]. Supervised machine learning includes extracting implicit, previously unexplored, and potentially valuable information from a given dataset. In other words, it is a process for data analysis toward pattern recognition and regularities [20]. Pattern recognition is the ability to recognize data arrangement and classification to provide insight into a given system or dataset [21]. In addition, the capacity to concurrently analyze numerical and categorical features is a prominent feature of supervised machine learning models. Adding categorical variables to predictive models opens up the opportunity to reduce the heterogeneity across different studies [22].

A decision tree is an efficient method for pattern discovery analysis in data mining algorithms [23]. It creates a diagram for visualizing data [20]. The performance or accuracy of trees in an unknown testing dataset is calculated after learning or training the prediction models. High accuracy or performance shows that the data are properly trained and can be applied for predicting future biomarkers [24].

In this study, machine learning methods, namely here decision tree (DT), gradient boosted tree (GBT), and RF, have been applied to analyze multiple datasets, which are combined in a meta-analysis from studies that evaluate gene expression in multiple cancer cell lines before and after different herbal extract treatments, focusing on differentially expressed transcription factors in the meta-analysis. Transcription factors were then used to train and build models to predict whether or not a sample belonged to the control or the treated group and identify a signature/pattern of transcription factors capable of discriminating between the groups.

## 2. Methods

**Figure 1:**
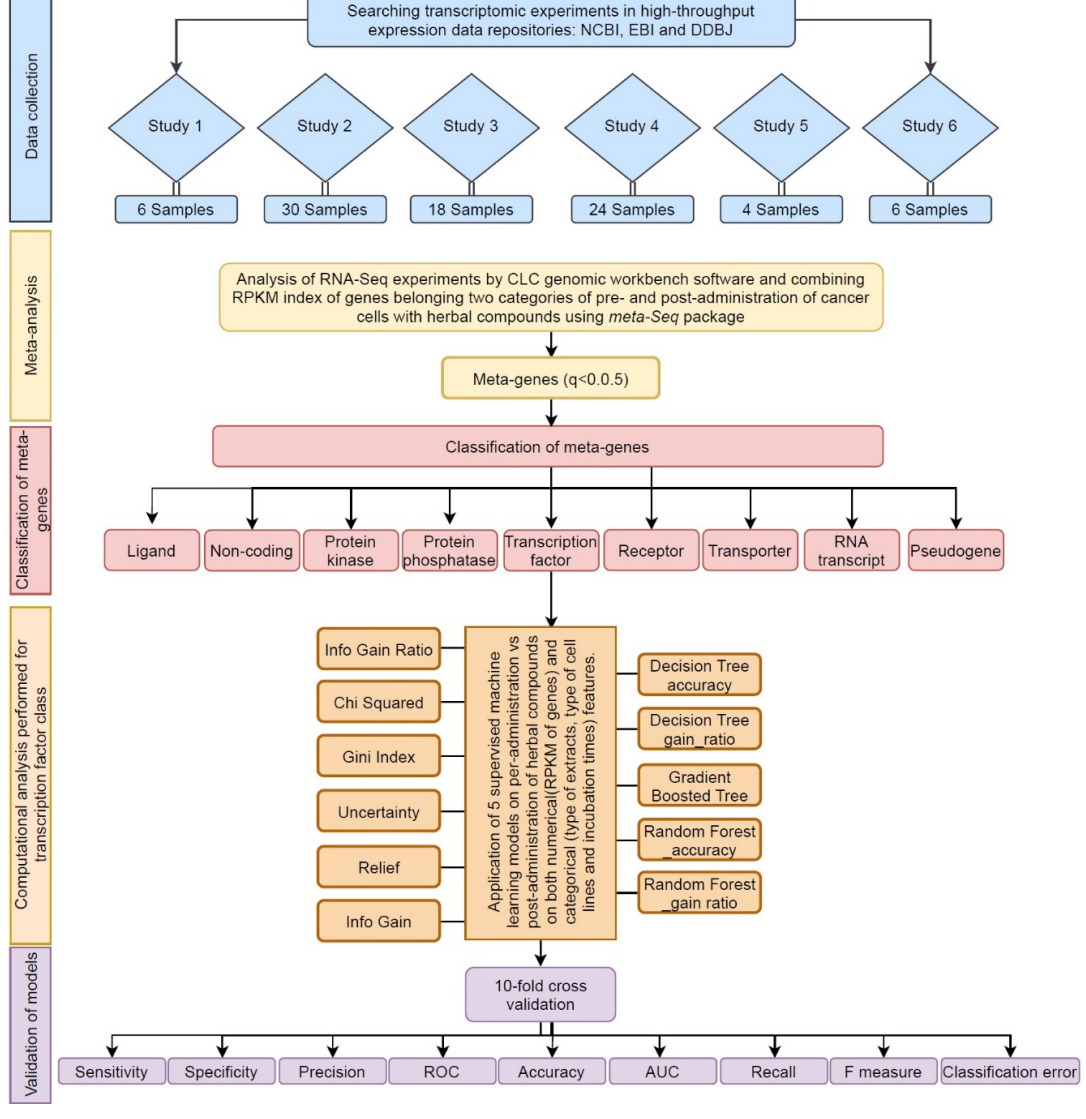
The flowchart of computational systems biological approach, developed in this study. This system’s biological approach includes 5 steps: data collection (blue), meta-analysis (yellow), meta-gene classification (red), pattern discovery (orange), and validation of model (purple).

The presented flowchart in Figure 1 illustrates the integrated method utilized in this study.

### 2.1. Data Collection

Three databases including SRA–NCBI (https://www.ncbi.nlm.nih.gov/sra accessed on 1 May 2020), EMBL-EBI (https://www.ebi.ac.uk/ena accessed on 1 May 2020), and DRA–DDBJ (https://www.ddbj.nig.ac.jp/dra/index-e.html accessed on 1 May 2020) were used as repositories of high-throughput expression. Datasets in connection with the effect of herbal compounds on inhibiting cancer cells were gathered for meta-analysis. Our target studies contained 88 samples: 36 control and 52 treated. Details of experiments are as follows: PCa treated via *Wedelia chinensis* extract (WCE) (concentration: 10 mg/mL/kg, incubation: 10 weeks) (GEO accession number GSE99820), HCT116, SW480, SW620, HT29, and RKO cell lines treated via oligomeric proanthocyanidins (OPC) from grape seeds and grape seed extract (GSE) (concentration: 100 ng/µL, incubation: 18 h) (GEO accession number GSE109607), A549 cell line treated via jinfukang (JFK) (concentration: 30 μg/mL, incubation: 48 h) (accession number ERP015444), MCF-7 cell line treated via compound kushen injection (CKI) (concentration: 1 and 2 mg/mL, incubation: 24 and 48 h) (GEO accession number GSE78512), LNCaP and PC-3 cell lines treated via Sulforaphane (SFN) (concentration: 15 µM, incubation: 6 and 24 h) (GEO accession number GSE48812), SK-BR-3, MCF-7, and MDA-MB-231 cell lines treated via shikonin (concentration: 10 μM, incubation: 6 h) (GEO accession number GSE100687). Other details about the collected datasets are represented in Table 1 and Table S1.

There were seven different types of herbal compounds within the six studies. The names and some details of them are as follows:

WCE is an extract of *Wedelia chinensis* herbal medicine. Several compounds including flavonoids, diterpenes, triterpene saponins, and phytosteroids were reported in this extract. Recently extensive studies have demonstrated the anti-cancer properties of WCE against prostate, lung, breast, colon, glioblastoma, and pancreatic cancer cells [25].

GSE is an extract made from the seeds of grapes and is reported to exhibit anti-cancer activity in several types of cancers. It contains a considerable amount of phenolic compounds such as epicatechin, catechin, procyanidins, and proanthocyanidins. Thus, it possesses excellent anti-oxidant properties [26].

OPC is abundantly found in grape seeds. OPC exhibited anti-proliferative activity and pro-apoptotic effect on prostate cancer. In addition, it could suppress the formation of tumors in colorectal cancer [27].

SFN is generally observed in numerous cruciferous vegetables, such as broccoli and cabbages. Several biological activities including anti-oxidant, anti-inflammatory, and anti-tumor actions have been reported for this [28,29].

Shikonin has been applied for multiple inflammatory and infectious diseases. Shikonin is a naphthoquinone extracted from the *Lithospermum erythrorhizon*, a Chinese medical herb. The pharmacological properties of shikonin are anti-bacterial, anti-virus, anti-oxidant, and anti-inflammatory activities. It has been illustrated that shikonin exerts anti-cancer effects through diverse mechanisms on different mitochondrial pathways in prostate, leukemia, and gastric cancers [30].

CKI is a classical medicine used in China for the clinical therapy of many kinds of cancers. CKI is isolated from the *Sophorae Flavescentis* and *Rhizoma smilacis Glabrae*. The biochemical analysis demonstrated that CKI comprises eight components, with primary two significant quinolizidine alkaloid compounds, including Matrine and Oxymatrine [31].

JFK is a Chinese medicine consisting of 12 Chinese medicinal plants and is mainly employed to treat lung cancer. The mechanisms of action of JFK are metastasis prevention and tumor lesion stabilization [32].

### 2.2. Meta-Analysis

Here, we employed RNA-Seq datasets of 6 individual investigations that were different in terms of extraction types, cancer types, incubation time, and also the dose of herbal compound (see Table 1). Due to the deficiency of RNA-Seq research on the impacts of herbal compounds against cancer cells, the studies were divided into 13 different levels. Each level was considered as an independent experiment. (Table 2 and Table S2).

*FASTQ* files of six RNA-Seq datasets were downloaded and analyzed using CLC genomics workbench software (version 11; CLC bio). Briefly, after finding the raw reads’ quality, those with low quality were trimmed off. Then, high-quality short reads were mapped into the human reference genome (hg19) using the following criteria: mismatch cost = 2, insertion cost = 3, deletion cost = 3, length fraction = 80%, and similarity fraction = 80%. RPKM index (reads per kilobase of transcript per million mapped reads) was reported as expression estimations for every gene and used as inputs for meta-analysis. Meta-analysis was implemented in R program (version 3.6.0) using the *Meta-Seq* package (version 1.22.1). This package uses *NOISeq* to detect genes that are differentially expressed. The number of reads is often different depending on the studies, and this generally influences statistical tests. *NOISeq* is almost not affected by the number of reads and helps overcome the read size effect bias [33].

Next, the overall statistical significance was calculated using Fisher’s probability test, and the identified genes were named meta-genes.

The obtained meta-genes were classified based on their function (using Pathway Studio Web Mammal, Elsevier, Amsterdam, The Netherlands). Finally, the TFs introduced by meta-analysis (named as meta-TFs) were considered for the following analysis.

### 2.3. Gene Ontology Analysis of Transcription Factors

DAVID classification system (http://david.abcc.ncifcrf.gov/home.jsp accessed on 10 October 2020) was used to obtain a complete set of the biological importance of meta-TFs. This database employs the *p*-value and Benjamini methods to determine the significance of pathways of input TFs.

### 2.4. Categorical Feature Analysis by Decision Tree Algorithms

For categorical feature analysis, a dataset containing 479 features of treated and control group was prepared. In this, RPKMs of meta-TFs were used as numerical features. Additionally, we added type of extracts, type of cell lines, and incubation time to the dataset as categorical features. Consequently, a dataset of 482 (479 RPKMs + type of extracts + type of cell lines + incubation time) and 88 records (samples) belonging to treatment and control categories (label variable) was prepared. Then, GBT, DT, and RF models were run on the dataset by rapidminer software (RapidMiner 9.7). DT and RF models contained two different criteria, including accuracy and gain ratio.

### 2.5. Validation and Comparison of Predictive Algorithms

In this study, trees were constructed using a ten-fold cross-validation algorithm to assess the models’ performance for predicting the correct class. To conduct ten-fold cross-validation, the dataset was partitioned into ten equal size sub-samples. The first nine samples were used as training sub-samples, and the last one was employed as unseen data. The cross-validation procedure was repeated ten times, and the average of the series was computed by dividing the percentage of accurate predictions over the total examples. Finally, accuracy, AUC, ROC, sensitivity, specificity, precision, recall, F measure, and classification error of models were determined.

### 2.6. Meta-Analysis of Individual Signature genes

Meta-analysis of individual signature genes was employed by combining the RPKM index for each gene. Effect size (mean difference between the RPKM in the treated vs. untreated control samples) was calculated separately for each of the 13 datasets. Fixed-effect and random-effect models were applied to determine overexpression of biosignature TFs in response to herbal compounds in carcinogenic cells. Additionally, the 95% confidence intervals (CI) were estimated. Positive and negative mean difference values showed upper and lower levels of gene expression. The forest plot was plotted to compare the mean differences of predictive genes in each independent dataset and overall effects for the selected genes. In addition, to determine whether prediction bias existed among different datasets, both Begg and Mazumdar rank correlation test and Egger’s test of the intercept were employed to examine the prediction bias on the summary estimates [34,35]. Non-significant *p*-value presenting the absence of publication bias. All analyses were performed with the Comprehensive Meta-Analysis 2.2 software.

### 2.7. External Validation for Effectiveness of the Predictive TFs on New Herbal Compound

For external validation to examine the effectiveness of the predictive TFs on the new herbal-derived compound, independent samples of treated and non-traded from an experiment with GEO accession of GSE40069 were selected. The original study was planned to investigate the effect of genistein on hepcidin expression in human hepatocytes [36]. Genistein is an isoflavone compound found in soy products. Many studies have proved its role in proliferation inhibiting and apoptosis-inducing in several carcinogenic cell types [37]. In the selected study, HepG2 cells were administered with the genistein (10 µM) for 18 h. In addition, samples with DMSO 1% were considered vehicle control. We selected three treated samples (GEO accessions: GSM984644, GSM984645, GSM984646) and three non-treated samples (GSM984647, GSM984648, and GSM984649) of this experiment. Raw SRA files of the selected samples (100 bp, paired-end, Illumina Genome Analyzer II sequencing technology) were downloaded and analyzed as described in Section 2.2. Finally, the differentially expressed genes were obtained.

## 3. Results

### 3.1. Increasing the Size of Dataset by Meta-Analysis

There was limited research available that assessed the effects of herbal compounds on cancer inhibition. Among the available datasets/data/studies in the transcriptomic database, six RNA-Seq datasets were selected.

The samples of eligible datasets which were administrated only with the herbal compound were allowed to be included in the meta-analysis. The total samples quantity was 88, which contained 36 pre-administration (control) and 52 post-administration (treatment) samples. Meta-analysis was implemented based on joining the RPKM index of 58,175 genes at 13 levels. As a result, 6992 meta-genes were upregulated differentially (one-tailed, q < 0.005), while no significant down-regulated genes were detected. Interestingly, 6180 meta-genes never revealed a significant *p*-value in any original studies, possibly due to the effect of inadequate replication on DEGs identification in single studies (see Table S3).

### 3.2. Classification of Meta-Genes

**Figure 2:**
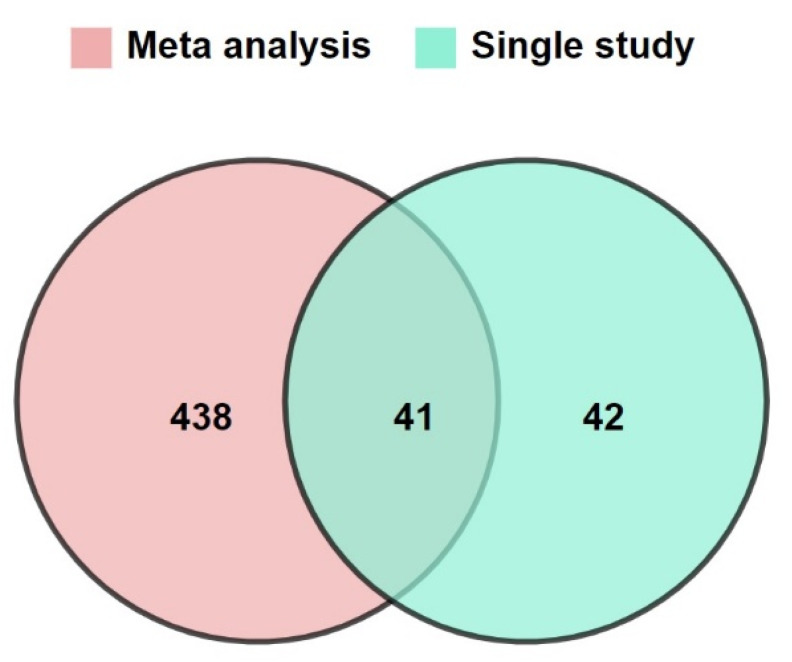
Venn diagram of differential expressed TFs showing the overlap between upregulated transcription factors identified by at least one study and the meta-analysis.

The meta-genes were classified into nine classes: ligand, non-coding, protein phosphatase, protein kinase, receptor, transcription factor, transporter, RNA transcript, and pseudogenes (Table S4). A total of 479 TFs that had significantly been changed in expression profile in response to herbal compounds were identified by meta-analysis at a cut-off *p*-value of 0.05. In comparison to the independent studies, the meta-analysis showed many TFs (438) that only were significantly different following this approach (Figure 2).

### 3.3. GO-Enrichment Analysis of Herbal-Induced TFs

**Figure 3:**
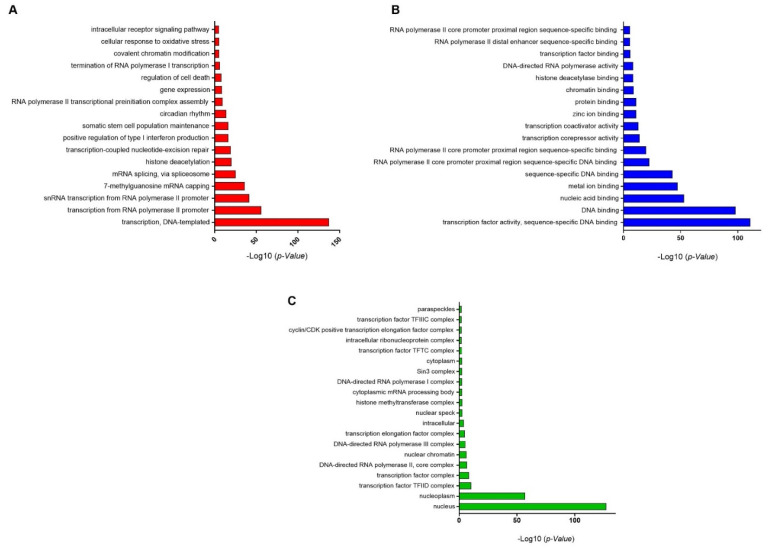
Gene ontology (GO) enrichment. Classification of the GO levels for upregulated transcription factors derived from a meta-analysis. Transcription factors were annotated in three classes: biological process (**A**), molecular function (**B**), and cellular component (**C**). The x-axis depicts -log (*p*-value), and y-axis shows GO terms.

All TFs were analyzed for their gene ontology (GO) terms and fold enrichment through the DAVID classification system using *Homo sapiens* as a reference. GO terms for molecular functions, biological processes, and cellular components were determined. In the biological process, transcription-DNA-templated, RNA polymerase II promoter and snRNA transcription from RNA polymerase II promoter were abundantly enriched (Figure 3A). In terms of molecular function, transcription factor activity, sequence-specific DNA binding, and other DNA binding were significantly enriched (Figure 3B). Regarding the cellular component class, genes were associated in response to the nucleus, nucleoplasm, transcription factor TFIID complex and transcription factor complex (Figure 3C). (Supplementary Table S5).

### 3.4. Discovery of Signature of Herbal Transcription Factors on Cancer Cells by Pattern Discovery

Five prediction algorithms (DT_gain ratio, DT_accuracy, GBT, RF_gain ratio, and RF_accuracy) were run on the TF dataset. Among the trained models, only RF trees (gain ratio and accuracy) could predict TF signatures correctly. The AUC, sensitivity, specificity, accuracy, precision, recall, F measure, and classification error criteria for RF models are presented below.

#### AUC

**Figure 4:**
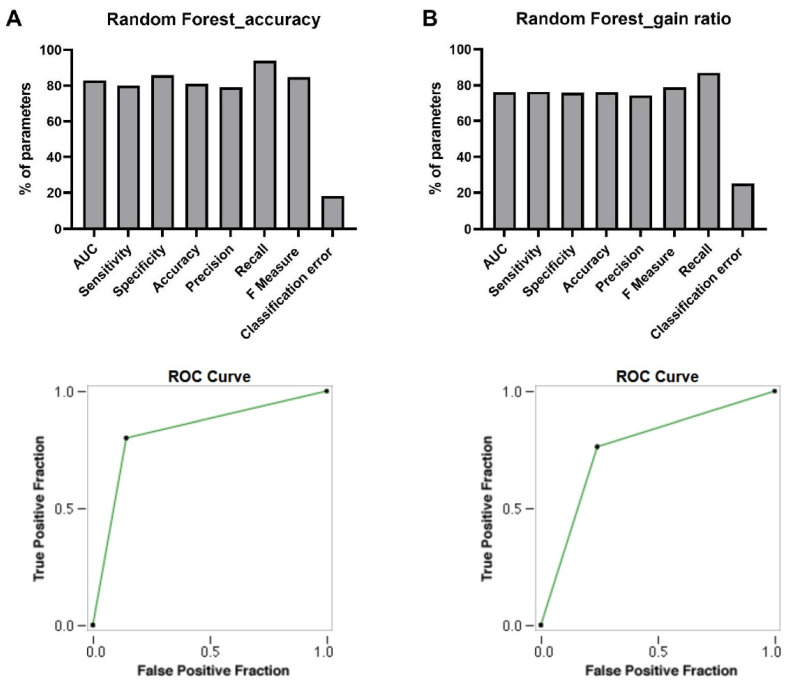
Comparison the performance of Random Forest accuracy (**A**) with Random Forest gain ratio (**B**).

As presented in Figure 4, RF_accuracy showed AUC value 0.829, and RF_gain ratio showed lower AUC values of 0.761 (Figure 4). RF_accuracy showed a higher AUC value, meaning this model is able to distinguish the positive class values (treated samples) from the negative class values (control samples) ideally.

#### Sensitivity

The sensitivity percentages of RF_accuracy and RF_gain ratio were 80 and 76.3, respectively (Figure 4). This showed 80% and 76.3% of the positive class points are classified correctly.

#### Specificity

RF_Accuracy model showed higher specificity in comparison with RF_gain ratio model. The specificity percentages of RF_Accuracy and RF_gain ratio were 85.7 and 76.3 respectively (Figure 4). This evaluation metric showed 85.7% and 76.3 % of the negative class was correctly classified.

#### Accuracy

The percentage of accuracy belonging to RF_accuracy was 81.8%, while the accuracy computed for the RF_gain ratio was 76.1% (Figure 4). Therefore, RF_accuracy model with an accuracy of 81.8% indicated that 72 out of 88 samples were correctly classified.

#### Precision

For TF prediction, the gained precisions of RF models were 79.9% and 74.27 % (Figure 4). Then, 79.9% and 74.27% of positive class predictions were actually part of the positive class.

#### Recall

In the prediction of TFs, the percentage of recall for the RF_accuracy model was 93.97% and for the RF_gain ratio was 86.85% (Figure 4). Based on the results of the recall metric, 93.97% and 86.85% positive class predictions were made out of all positive examples in the dataset.

#### F Measure

The F measure criteria were 84.85% and 78.78 % for RF_accuracy and RF_gain ratio, respectively (Figure 4). This is an appropriate criterion for assessing the accuracy of models and considers both precision and recall in one number.

#### Classification Error

The classification errors for the RF_gain ratio and RF_accuracy were 25.3% and 18.17%. (Figure 4).

#### ROC

RF_accuracy showed the best area under ROC curve in predicting the true-positive rate versus false-positive rate, supporting it as a strong model for TF signatures prediction (Figure 4).

More details of different decision tree models in predicting transcription factor signatures were presented in Supplementary file 1.

**Figure 5:**
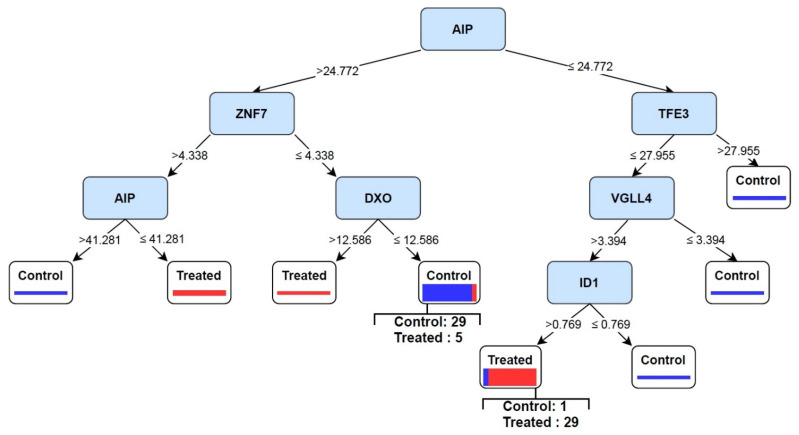
The AIP/TFE3/VGLL4/ID1 pattern was induced in 29 treated samples and one control case. In contrast, the AIP/ZNF7/DXO pattern was observed in 29 control and five treated samples.

**Figure 6:**
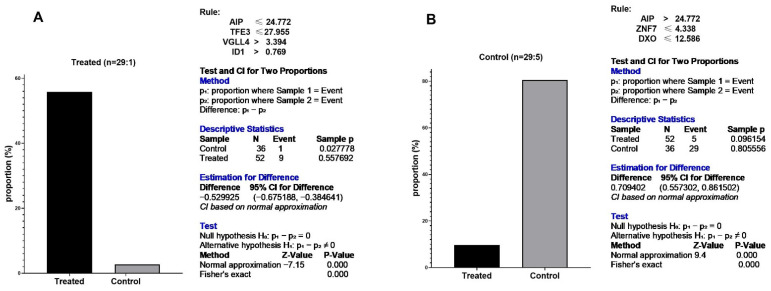
Regulatory signatures of herbal treatments on carcinogenic cells. (**A**): AIP/TFE3/VGLL4/ID1 rule induced in 55.7% of treated and 2.7% of control cases. (**B**): AIP/ZNF7/DXO rule was observed in 80.5% of control and 9.6% of treated samples. Proportion test by Fisher Exact test confirms the results of Random Forest where most of cells obey the rules belong to cancer.

As shown in Figure 5, two rules were obtained based on the RF model. AIP/TFE3/VGLL4/ID1 rule was observed in treated samples, and AIP/ZNF7/DXO rule was demonstrated in control cases. The proportion test showed obtained rules were significantly induced in each group (Figure 6). AIP is the most important feature setting at the top of the tree. If TF values were greater than 24.772, the samples fell into the control group. In contrast, samples fell into the treated category if the values were equal to or smaller than 24.772. Therefore, this model becomes an excellent candidate to separate control and treated samples and has the potential biomarker performance.

### 3.5. Predictive Signature Genes between Treated and Control Samples Are Corroborated by Individual Gene Meta-Analysis

**Figure 7:**
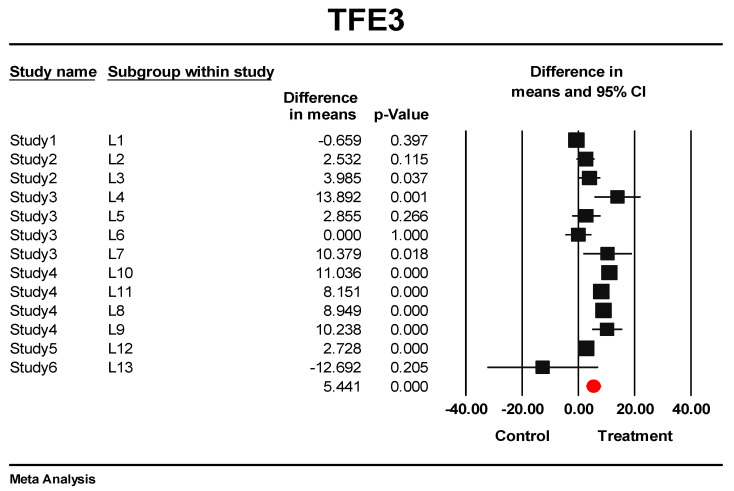
RPKM meta-analysis of thirteen datasets for overall expression of TFE3 in treated and control groups. The black square shows the mean difference of treated and control RPKMs for each study. The red circle shows the summary of the overall effect size across all studies. The effect size direction is higher than zero if the expression is higher in treated samples.

Meta-analysis of AIP, TFE3, VGLL4, and ID1 RPKM was performed using thirteen datasets (Table 2). According to the results, the fixed model indicated significant upregulation for all genes (*p* < 0.001) (see Figure 7 for TFE3; the forest plot of AIP, VGLL4, and ID1 are presented in Supplementary Figures S1–S4). The Random model also was significant for upregulation of all genes (*p* ≤ 0.01) except ID1 (*p* = 0.210). For most of the datasets, when analyzed individually, the genes seem not to reach significant over-expression, but when the meta-analysis of the datasets was performed, statistical significance was obtained. This proves the meta-analysis’s power in providing statistically significant results. In addition, Begg and Mazumdar rank correlation test and Egger’s test of the intercept showed that no obvious prediction bias existed in the individual meta-analysis of genes (Table 3). It indicates that the estimated effect size for significant expression of the signature genes in treated samples was not affected by a dataset. These findings confirm the validity and generalization of predicted genes.

### 3.6. Eternal Validation of AIP, TFE3, VGLL4, and ID1

A dataset corresponding to purely one herbal compound that was not involved in differential analysis and pattern recognition used to examine the effectiveness of the predictive TFs on new herbal medicine. Results showed the regulatory signature, derived from meta-analysis and machine learning combination, were repeatable when a new and independent experiment was utilized for its validation (Table 4).

## 4. Discussion

Considering the medicinal importance of plant-derived compounds, the development of new anti-cancer drugs based on plants is still the primary strategy in the modern drug discovery era [1]. Identifying potential genes that are targeted by plant compounds is a critical step in the drug discovery process [38]. Recently, the accumulation of large-scale NGS data has tailored computational methods such as machine learning and meta-analysis to discover key genes and biomarkers which govern biological effects [39]. Thus, this study was focused on detecting novel TFs as predictive biomarkers associated with herbal components in cancer cell lines using a combination of meta-analysis and machine learning models.

Meta-analysis is a way to combine the results of independent studies, which increases the sample size and improves the results’ generalizability and statistical capability [18]. Additionally, supervised machine learning models can simultaneously analyze both categorical and numerical features, and therefore, they have brought in a good possibility to discover patterns within any given datasets and predict many events out of available data [17]. Adding categorical variables to predictive models reduced the heterogeneity across studies, in agreement with previous findings [23]. In this study, the type of extract, type of cell line, and incubation times were considered as categorical features. Therefore, the analysis includes treatment effect as well as cell type, extract type, and incubation times. Adding more confounding factors as categorical variables contributes to increasing the accuracy of models.

The meta-analysis was able to successfully identify 6992 upregulated DEGs which included 479 TFs. Based on the biological process term from GO results, the identified meta-TFs were significantly involved in regulating RNA polymerase II. As all meta-TFs were upregulated during herbal-compound treatments, the expression of genes involved in controlling transcription from RNA polymerase II promoter was required. In addition, according to GO annotation for molecular function, a large number of meta-TFs classified into the binding category, which is because of TFs’ ability in binding to the promoter region.

Based on categorical feature analysis, the best patterns were obtained through the RF_accuracy model. The AIP/TFE3/VGLL4/ID1 pattern was upregulated in treated cases, whereas AIP/ZNF7/DXO pattern was enhanced in control samples. Previous studies showed that the RF model offers the most reliable accuracy in numerous scientific fields of study in recent years [40]. That is because it uses the power of several trees (100 trees in this study) for training data and making decisions. Each node in this model runs on a random subset to compute the output. Finally, the outputs of all individual models are combined to generate the overall outcome. In consequence, the random forest concludes the data in a safer procedure. This randomized feature selection makes the random forest much more accurate than other decision trees [41]. For this reason, the biomarkers introduced in this study were highlighted as the ideal candidate to evaluate the effectiveness of herbal compound on a particular cell. 

In this study, AIP was identified as the most important biomarker. AIP is an immunophilin-like protein ubiquitously expressed in the cytoplasm [42]. AIP contributes to cell growth regulation by mediating cell-cycle factors including p27^Kip1^, p18^Ink4c^, and Rb. Raitila et al. used murine models and in vitro studies to investigate the anti-tumor role of AIP. Based on their results, the proliferation of pituitary cells was decreased in AIP overexpressed samples. By contrast, in AIP knock-down samples, cell proliferation was increased [43]. These findings confirmed the tumor suppressor action of AIP in pituitary adenomas. Moreover, several types of research suggested that AIP can inhibit tumor formation via regulation of cell division and cAMP signaling cascade [44,45]. Increasing cAMP signaling represents a mitogenic signal for the somatotroph cell. AIP decreased the subcellular concentration of cAMP, and its deficiency elevated the intracellular cAMP concentration in pituitary cell lines. The results suggested that AIP might contribute to the tumor suppressor effects by inhibiting the cAMP pathway [42,46]. It has also been suggested that AIP might regulate the expression of p27, which is an important negative regulator of the cell cycle [42].

TFE3, the second gene belonging to the treated rule, is a master transcriptional regulator of several biological processes, including autophagy, inflammatory process, and the unfolded protein response [47]. Ample evidence showed TFE3 directly upregulates the p53 tumor suppressor gene [48]. P53 is an essential regulator of the DNA damage response and controls the transcription of many downstream genes involved in DNA repairing, arresting the cell cycle, and inducing apoptosis. It has been shown that p53 activates TFE3 via its negative effects on mTORC1 action in response to DNA damage [48]. Through two mechanisms, including feedback and feedforward controls, p53-dependent activation of TFE3 promotes stabilization and protein levels of p53. Therefore, it is proposed that TFE3 facilitates apoptosis in response to prolonged DNA damage [48].

Another biomarker found by the treated rule was VGLL4. Vestigial like family member 4 is a transcriptional cofactor from the VGLL family. In contrast to other VGLL family members, VGLL4 works as a novel tumor suppressor through cooperating with TEAD transcription factors [49]. A considerable number of works in literature have evidenced that the expression of VGLL4 is significantly weaker compared to healthy tissues in many kinds of cancers [50,51,52,53,54,55,56]. VGLL4 has a critical function in several signaling pathways. For example, in Salvador/Warts/Hippo (SWH) signaling pathway, there is competitive interaction between VGLL4 and YAP in coupling with TEADs. The combination of YAP and TEAD accelerates cell proliferation and inhibits apoptosis [49,57]. Conversely, binding VGLL4 to TEADs suppresses the expression of the downstream oncogenes [56]. On the other hand, VGLL4, via restricting β-catenin and T-cell factors, negatively control the Wnt/β-catenin signaling pathway. VGLL4 can also overcome epithelial-mesenchymal transition and commit to the apoptosis signaling pathway [52].

ID1 (Inhibitor of differentiation/DNA binding 1) is an oncogenic protein. However, recently, it has been suggested that this gene may play a role in increasing drug sensitivity of non-small cell lung cancer (NSCLC). Upregulation of ID1 was connected with helpful prognoses for patients administered with adjuvant paclitaxel plus cisplatin after surgery. Tan et al. used murine orthotropic lung carcinoma models with or without stable ID1 overexpression. The murine models were treated with gefitinib. The results showed that upregulation of ID1 in gefitinib-treated NSCLC cells induced necroptosis. Hence, they concluded that ID1 could elevate NSCLC cells’ sensitivity to gefitinib [58].

According to the obtained tree in Figure 5, ZNF7 and DXO were upregulated in the control rule. Emerging studies have demonstrated that ZNF7 is critical for inhibiting TNF-α-mediated apoptosis by A20 [59].

Few studies have investigated the biological role of DXO (Decapping and exoribonuclease protein) in cancers. DXO regulates several processes linked to mRNA 5′-end capping, including decapping, pyrophosphohydrolase, deNADding, and 5′–3′ exoribonuclease activities. It has been revealed that cell proliferation was increased by DXO downregulation and destabilizing cyclin D1 mRNA in bladder cancer. However, genes controlled by this transcription factor have not yet been identified, indicating that more research is required to be performed [60].

There are some limitations in this study, including a limited number of available studies on the effectiveness of plant-derived compounds on cancers. We employed an integrated approach of meta-analysis and machine learning to aggregate the different datasets from different cell lines. The following strategies were employed to minimize the batch effects: (1) RPKM index was used as expression measurement of genes in meta-analysis. RPKM normalizes the counts of the mapped reads to a gene in respect to the transcript length and the sequencing depth. Consequently, expression measurements across different genes and different experiments were comparable [61]. (2) All experiments included in this study used the illuminia platform of sequencing (Table S1). (3) Meta-analysis was conducted by the *metaSeq* package of R, which is a robust method against read-size effect and also uses TMM normalization [33].

## 5. Conclusions

This study was designed to discover a core set of transcription factors responding to different herbal compounds in various carcinogenic cells. First, 479 differentially upregulated TFs were detected by meta-analysis. Then, the best combination of TFs/features that accurately discriminated herbal-treated samples from untreated ones was determined via categorical feature analysis. The results showed that the machine learning method combined with meta-analysis successfully identified general TFs responding to herbal-derived compounds. RF model with accuracy criterion performed better in mining important transcription factors. This tree also provided a more effective and reproducible bio-signature. The reason is that the RF algorithm merges the yield of various random trees to generate the final result. The ability of random feature selection makes RF considerably more accurate in comparison to other models. As a result, the identified biomarkers in this study might be ideal candidates to distinguish whether an investigational new herbal compound is effective on a particular cell or not. In addition, reported results in this article confirm machine-based prediction’s capability in finding the relation between important transcription factors.

## Figures and Tables

**Table 1:** Studies and samples employed in this investigation to find a regulatory signature of transcription factors responding to different herbal compounds in various carcinogenic cells.

Study	Reference	Accession of Experiment	No. of Arrays (Control: Treatment)	Organism	Cell Line(s)	Herbal Treatment	Incubation Time	Dose of Compound	Platform
1	PMC:5688072	GSE99820	6 (3:3)	*Homo sapiens*	PCa	*Wedelia chinensis* extract (WCE)	10 weeks	10 mg/mL/kg	Illumina HiScanSQ
2	PMID: 29463813	GSE109607	30 (10:20)	*Homo sapiens*	HCT116, SW480, SW620, HT29, RKO	Oligomeric proanthocyanidins (OPC) Grape seed extract (GSE)	18 h	100 ng/µL	Illumina HiSeq 2500
3	PMID: 27602759	GSE78512	24 (12:12)	*Homo sapiens*	MCF-7	Compound Kushen Injection (CKI)	24 and 48 h	1 mg/mL and 2 mg/mL	Illumina HiSeq 2500 (*Homo sapiens*)
4	PMID: 25044704	GSE48812	36 (12:24)	*Homo sapiens*	LNCaP, PC3	Sulforaphane (SFN)	6 and 24 h	15 μM	Illumina HiSeq 2000
5	PMID: 28771580	ENA-ERP010522	4 (2:2)	*Homo sapiens*	A549	Jinfukang (JFK)	48 h	30 μg/mL	Illumina HiSeq 2000
6	PMID:29422643	GSE100687	6 (3:3)	*Homo sapiens*	MCF-7, SK-BR-3, MDA-MB-231	shikonin	6 h	10 μM	Illumina HiSeq 2500

**Table 2:** Six studies were divided into 13 different levels.

No. of Levels	Study	Cell Line	Extract	Time	Concentration
1	1	PCa	Wedelia Chinensis Extract (WCE)	10 weeks	10 mg/mL/kg
2	2	HCT116, HT29,RKO, SW480, SW620	Grape Seed Extract (GSE)	18 h	100 ng/µL
3	2	HCT116, HT29, RKO, SW480,SW620	Oligomeric Proanthocyanidins (OPC)	18 h	100 ng/µL
4	3	MCF-7	Compound Kushen Injection (CKI)	24 h and 48 h	1 mg/mL
5	3	MCF-7	Compound Kushen Injection (CKI)	24 h and 48 h	2 mg/mL
6	3	MCF-7	Compound Kushen Injection (CKI)	24 h	1 and 2 mg/mL
7	3	MCF-7	Compound Kushen Injection (CKI)	48 h	1 and 2 mg/mL
8		PC-3	Sulforaphane (SFN)	6 h and 24 h	15 µM
9	4	LNCAP	Sulforaphane (SFN)	6 h and 24 h	15 µM
10	4	PC3, LNCAP	Sulforaphane (SFN)	6 h	15 µM
11	4	PC3, LNCAP	Sulforaphane (SFN)	24 h	15 µM
12	5	A549	Jinfukang (JFK)	48 h	30 µg/mL
13	6	MCF-7, SK-BR-3, MBDA-MB-231	Shikonin	6 h	10 µM

**Table 3:** Prediction bias indices for each signature TFs.

Begg and Mazumdar Rank Correlation					Egger’s Regression Intercept			
TFs	Tau	z-Value for Tau	*p*-Value (1 Tailed)	*p*-Value (2-Tailed)	Intercept	Standard Error	*p*-Value (1-Tailed)	*p*-Value (2-Tailed)
AIP	0.25641	1.22018	0.11120	0.22240	−1.81915	1.72920	0.15766	0.31533
TFE3	0.02546	0.12202	0.45144	0.90288	1.30813	1.64424	0.22155	0.44311
VGLL4	0.10256	0.48807	0.31275	0.62550	1.6753	1.15997	0.16790	0.33579
ID1	0.28205	1.34220	0.08997	0.17953	−170452	0.98115	0.055511	0.11022

**Table 4:** Differentially expression of AIP, VGLL4, TFE3, and ID1 in response to genistein in HepG2 cell line.

GeneID	logFC	logCPM	F	*p*-Value	FDR
AIP	2.761854	6.441737	53.73853	2.31 × 10^−1^^3^	1.08 × 10^−11^
VGLL4	0.868611	3.701739	5.621917	0.017739	0.065989
TFE3	1.695222	5.920831	22.33107	2.30 × 10^−6^	2.46 × 10^−5^
ID1	2.212446	7.433711	33.69819	6.45 × 10^−9^	1.22 × 10^−7^

## Data Availability

The results shown here are based upon data generated by the GSE99820, GSE109607, GSE78512, GSE48812, ERP015444, GSE100687.

## Supplementary Materials

The following are available online at https://www.mdpi.com/article/10.3390/cells10113139/s1, Table S1: The detail of collected RNA-Seq studies used for meta-analysis, Table S2: RPKM indexes of 58175 genes in 13 different levels, Table S3: The results of meta-analysis, Table S4: List of classified genes, Table S5: Go enrichment details for TFs for molecular function, biological process, and cellular component terms, Supplementary file 1: Results of different machine learning models in predicting transcription factor signatures, Figure S1: RPKM meta-analysis of thirteen datasets for overall expression of AIP in treated and control groups, Figure S2: RPKM meta-analysis of thirteen datasets for overall expression of VGLL4 in treated and control groups, Figure S3 and S4: RPKM meta-analysis of thirteen datasets for overall expression of ID1 in treated and control groups.
