# Supplementary material for: Evaluation of the Effectiveness of Herbal Components Based on Their Regulatory Signature on Carcinogenic Cancer Cells
Source: Cells. 2021 Nov 12;10(11):3139. doi: 10.3390/cells10113139 (PMC8621084; doi:10.3390/cells10113139)
Supplement: Supplementary file 1 [file cells-10-03139-s001.zip › cells-1423536-supplementary/cells-1423536-supplementary.pdf]

AIP

Study name	Subgroup within study	Difference in means	p-Value
Study1	L1	-0.799	0.725
Study2	L3	-1.059	0.446
Study2	L2	0.596	0.669
Study3	L6	25.174	0.000
Study3	L7	-15.284	0.000
Study3	L4	9.988	0.204
Study3	L5	4.713	0.621
Study4	L9	2.787	0.025
Study4	L8	2.547	0.229
Study4	L10	-3.175	0.465
Study4	L11	2.338	0.619
Study5	L12	12.213	0.000
Study6	L13	3.528	0.660
		5.308	0.000

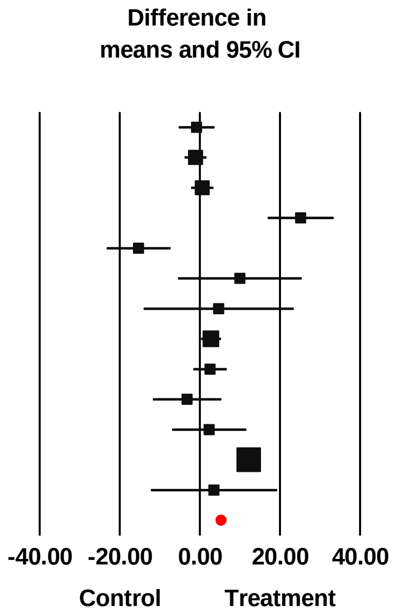

Meta Analysis

Figure S1. RPKM meta-analysis of thirteen datasets for overall expression of AIP in treated and control groups. The forest plot shows the differences in RPKM levels. The red circle shows the summary of the overall Fixed-effect and Random-effect sizes across all studies. The effect size direction is higher than zero if the expression is higher in treated samples.

VGLL4

Study name	Subgroup within study	Difference in means	p-Value
Study1	L1	0.946	0.412
Study2	L3	1.653	0.353
Study2	L2	1.954	0.315
Study3	L7	4.268	0.000
Study3	L6	1.715	0.199
Study3	L5	2.986	0.004
Study3	L4	3.092	0.001
Study4	L9	0.898	0.033
Study4	L8	-1.187	0.014
Study4	L11	-1.020	0.165
Study4	L10	0.730	0.029
Study5	L12	0.640	0.203
Study6	L13	-1.792	0.631
		0.764	0.000

Difference in means and 95% CI

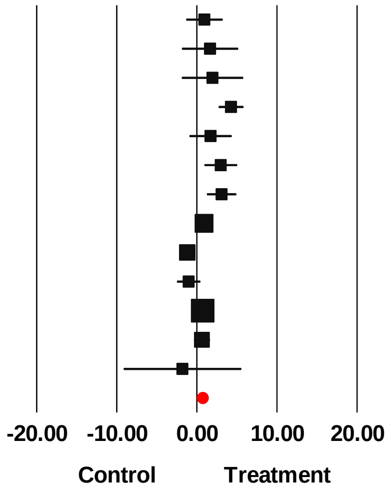

Meta Analysis

Figure S2. RPKM meta-analysis of thirteen datasets for overall expression of VGLL4 in treated and control groups. The forest plot shows the differences in RPKM levels. The red circle shows the summary of the overall Fixed-effect and Random-effect sizes across all studies. The effect size direction is higher than zero if the expression is higher in treated samples.

ID1

Study name	Subgroup within study	Difference in means	p-Value
Study1	L1	-14.405	0.013
Study2	L2	-2.008	0.812
Study2	L3	-6.678	0.377
Study3	L4	-5.104	0.122
Study3	L5	-10.232	0.054
Study3	L6	-2.419	0.442
Study3	L7	8.471	0.024
Study4	L10	56.383	0.000
Study4	L11	26.961	0.000
Study4	L8	28.370	0.191
Study4	L9	54.974	0.130
Study5	L12	14.285	0.000
Study6	L13	-9.214	0.659
		5.285	0.210

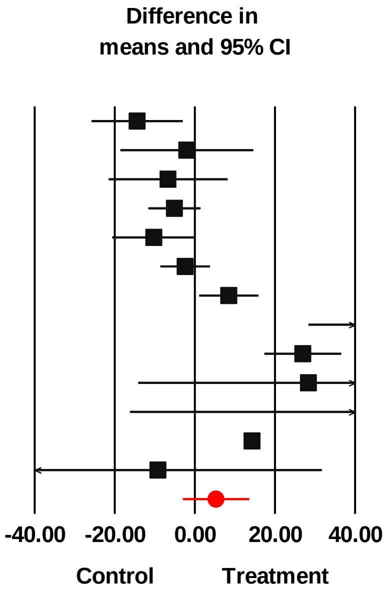

Meta Analysis

Figure S3. RPKM meta-analysis of thirteen datasets for overall expression of ID1 in treated and control groups. The forest plot shows the differences in RPKM levels. The center of the circle shows the summary of the overall Random-effect size across all studies. The effect size direction is higher than zero if the expression is higher in treated samples.

ID1

Study name	Subgroup within study	Difference in means	p-Value
Study1	L1	-14.405	0.013
Study2	L2	-2.008	0.812
Study2	L3	-6.678	0.377
Study3	L4	-5.104	0.122
Study3	L5	-10.232	0.054
Study3	L6	-2.419	0.442
Study3	L7	8.471	0.024
Study4	L10	56.383	0.000
Study4	L11	26.961	0.000
Study4	L8	28.370	0.191
Study4	L9	54.974	0.130
Study5	L12	14.285	0.000
Study6	L13	-9.214	0.659
		13.463	0.000

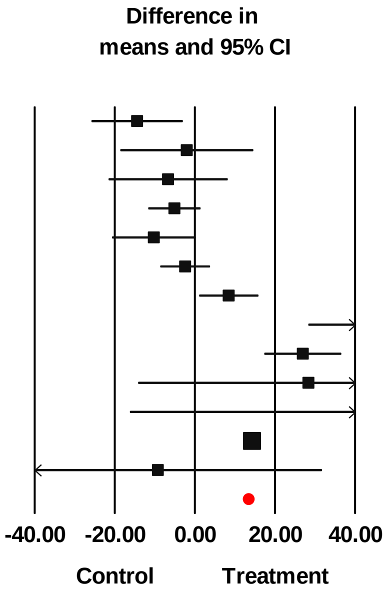

Meta Analysis

Figure S4. RPKM meta-analysis of thirteen datasets for overall expression of ID1 in treated and control groups. The center of the circle shows the summary of the overall Fixed-effect size across all studies. The effect size direction is higher than zero if the expression is higher in treated samples.
