# Supplementary material for: Evaluation of the Effectiveness of Herbal Components Based on Their Regulatory Signature on Carcinogenic Cancer Cells
Source: Cells. 2021 Nov 12;10(11):3139. doi: 10.3390/cells10113139 (PMC8621084; doi:10.3390/cells10113139)
Supplement: Supplementary file 1 [file cells-10-03139-s001.zip › cells-1423536-supplementary/Supplementary File 1/DT_accuracy/DT_accuracy.docx]

# Tree

EXTRACT = Control: Control {Control=36, Treated=0}

EXTRACT = GSE: Treated {Control=0, Treated=10}

EXTRACT = JFK: Treated {Control=0, Treated=2}

EXTRACT = Kushen: Treated {Control=0, Treated=12}

EXTRACT = OPC: Treated {Control=0, Treated=10}

EXTRACT = SFN: Treated {Control=0, Treated=12}

EXTRACT = Shikonin: Treated {Control=0, Treated=3}

EXTRACT = WCE: Treated {Control=0, Treated=3}
