# Supplementary material for: Evaluation of the Effectiveness of Herbal Components Based on Their Regulatory Signature on Carcinogenic Cancer Cells
Source: Cells. 2021 Nov 12;10(11):3139. doi: 10.3390/cells10113139 (PMC8621084; doi:10.3390/cells10113139)
Supplement: Supplementary file 1 [file cells-10-03139-s001.zip › cells-1423536-supplementary/Supplementary File 1/GB/GB.docx]

# Tree

Extract in {Kushen,Shikonin,SFN,... (4 more)}: 0.017 {}

Extract not in {Kushen,Shikonin,SFN,... (4 more)}: -0.024 {}
