# Supplementary material for: Evaluation of the Effectiveness of Herbal Components Based on Their Regulatory Signature on Carcinogenic Cancer Cells
Source: Cells. 2021 Nov 12;10(11):3139. doi: 10.3390/cells10113139 (PMC8621084; doi:10.3390/cells10113139)
Supplement: Supplementary file 1 [file cells-10-03139-s001.zip › cells-1423536-supplementary/Supplementary File 1/RF_accuracy/RF_accuracy.docx]

**Tree**

AIP > 24.772

| ZNF7 > 4.338

| | AIP > 41.281: Control {Control=2, Treated=0}

| | AIP ≤ 41.281: Treated {Control=0, Treated=9}

| ZNF7 ≤ 4.338

| | DXO > 12.586: Treated {Control=0, Treated=6}

| | DXO ≤ 12.586: Control {Control=29, Treated=5}

AIP ≤ 24.772

| TFE3 > 27.955: Control {Control=3, Treated=0}

| TFE3 ≤ 27.955

| | VGLL4 > 3.394

| | | ID1 > 0.769: Treated {Control=1, Treated=29}

| | | ID1 ≤ 0.769: Control {Control=1, Treated=0}

| | VGLL4 ≤ 3.394: Control {Control=3, Treated=0}
