# Supplementary material for: Evaluation of the Effectiveness of Herbal Components Based on Their Regulatory Signature on Carcinogenic Cancer Cells
Source: Cells. 2021 Nov 12;10(11):3139. doi: 10.3390/cells10113139 (PMC8621084; doi:10.3390/cells10113139)
Supplement: Supplementary file 1 [file cells-10-03139-s001.zip › cells-1423536-supplementary/Supplementary File 1/RF_gain ratio/RF_gain ratio.docx]

# Tree

DRAP1 > 26.011

| HMGN4 > 39.471: Control {Control=6, Treated=0}

| HMGN4 ≤ 39.471

| | VGLL4 > 3.379

| | | JMJD4 > 13.810: Control {Control=3, Treated=0}

| | | JMJD4 ≤ 13.810

| | | | JUN > 9.217

| | | | | ZNF79 > 10.435: Control {Control=2, Treated=0}

| | | | | ZNF79 ≤ 10.435

| | | | | | ZSCAN29 > 6.251

| | | | | | | SFN > 44.658: Treated {Control=0, Treated=1}

| | | | | | | SFN ≤ 44.658: Control {Control=1, Treated=0}

| | | | | | ZSCAN29 ≤ 6.251

| | | | | | | NONO > 100.926

| | | | | | | | RFXANK > 13.369: Control {Control=4, Treated=0}

| | | | | | | | RFXANK ≤ 13.369: Treated {Control=0, Treated=10}

| | | | | | | NONO ≤ 100.926: Treated {Control=0, Treated=47}

| | | | JUN ≤ 9.217: Control {Control=4, Treated=0}

| | VGLL4 ≤ 3.379: Control {Control=4, Treated=0}

DRAP1 ≤ 26.011: Control {Control=6, Treated=0}
