# Supplementary figures and images for: Evaluation of the Effectiveness of Herbal Components Based on Their Regulatory Signature on Carcinogenic Cancer Cells
Source: Cells. 2021 Nov 12;10(11):3139. doi: 10.3390/cells10113139 (PMC8621084; doi:10.3390/cells10113139)

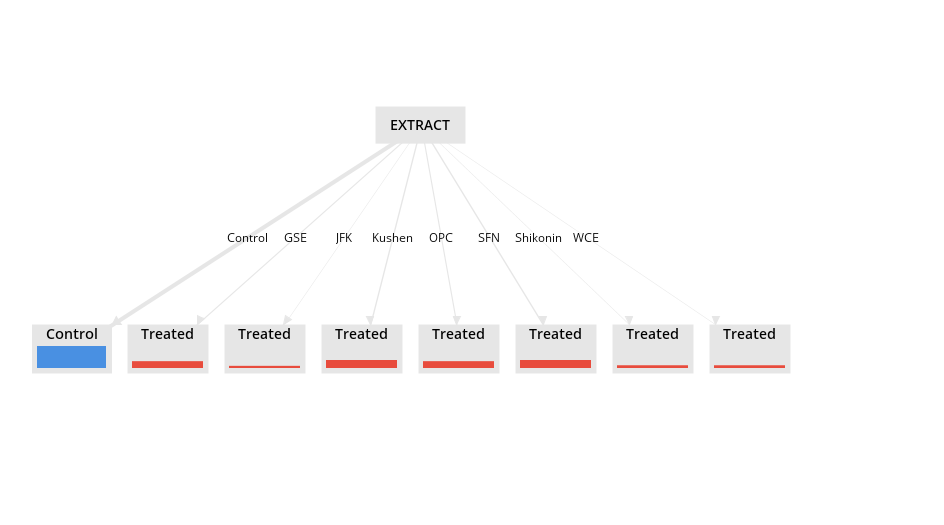

Supplement: Supplementary file 1 [file cells-10-03139-s001.zip › cells-1423536-supplementary/Supplementary File 1/DT_accuracy/DT_accuracy.png]

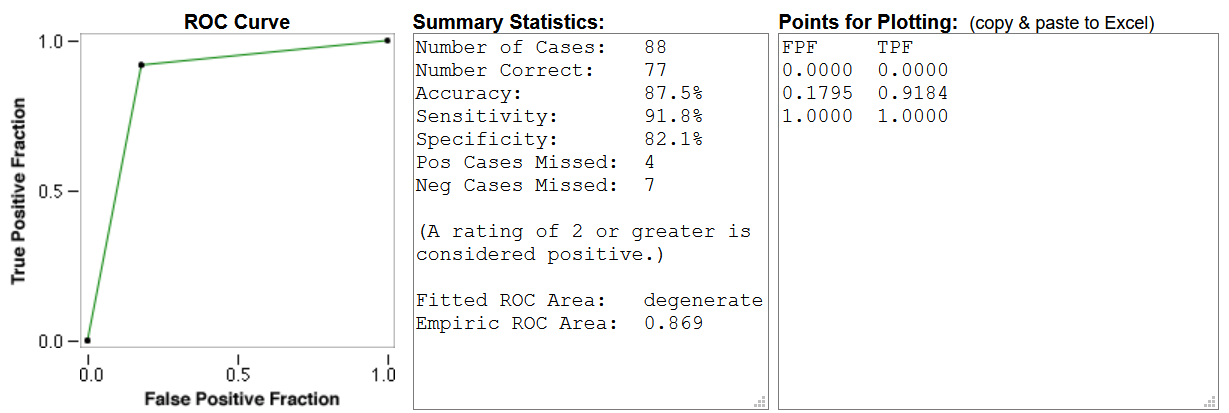

Supplement: Supplementary file 1 [file cells-10-03139-s001.zip › cells-1423536-supplementary/Supplementary File 1/DT_accuracy/ROC DT_accuracy.png]

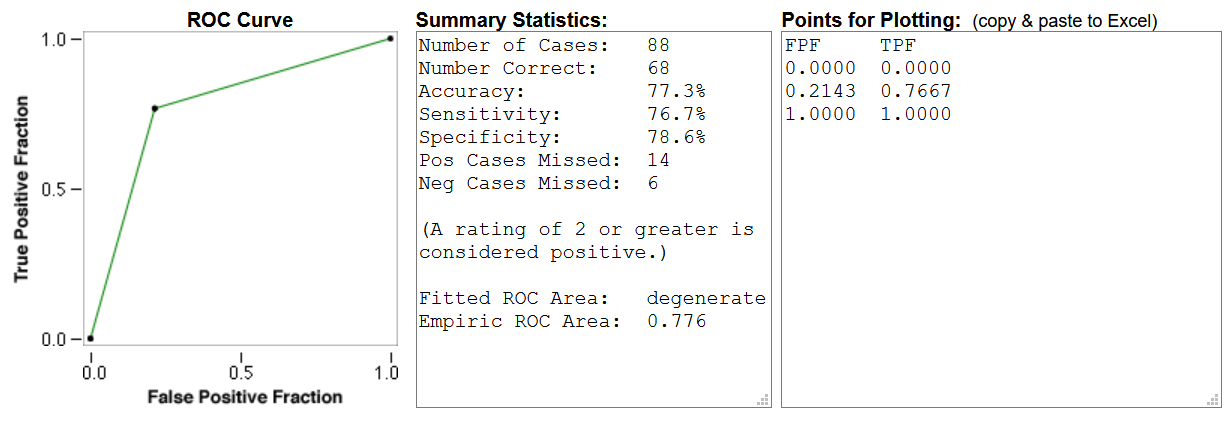

Supplement: Supplementary file 1 [file cells-10-03139-s001.zip › cells-1423536-supplementary/Supplementary File 1/DT_gain ratio/ROC DT_gainratio.png]

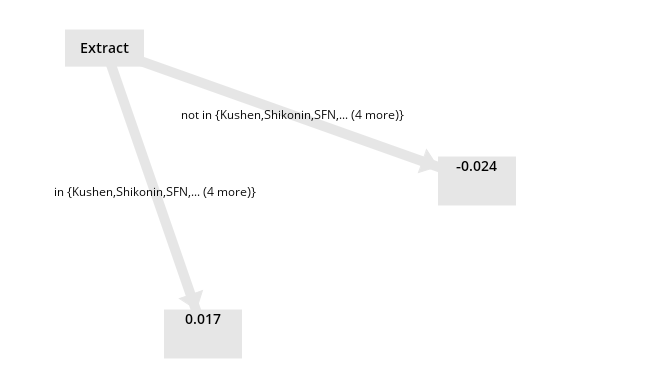

Supplement: Supplementary file 1 [file cells-10-03139-s001.zip › cells-1423536-supplementary/Supplementary File 1/GB/GB.png]

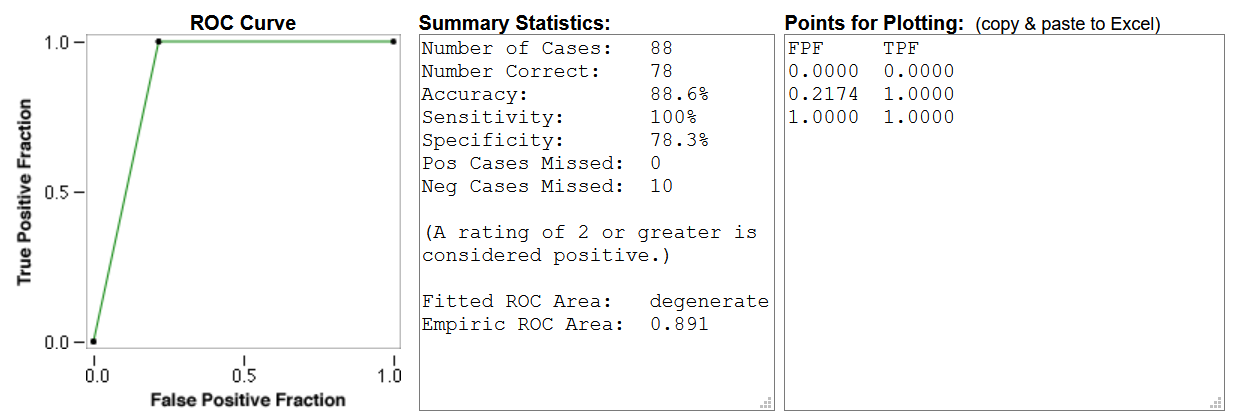

Supplement: Supplementary file 1 [file cells-10-03139-s001.zip › cells-1423536-supplementary/Supplementary File 1/GB/ROC GB.png]

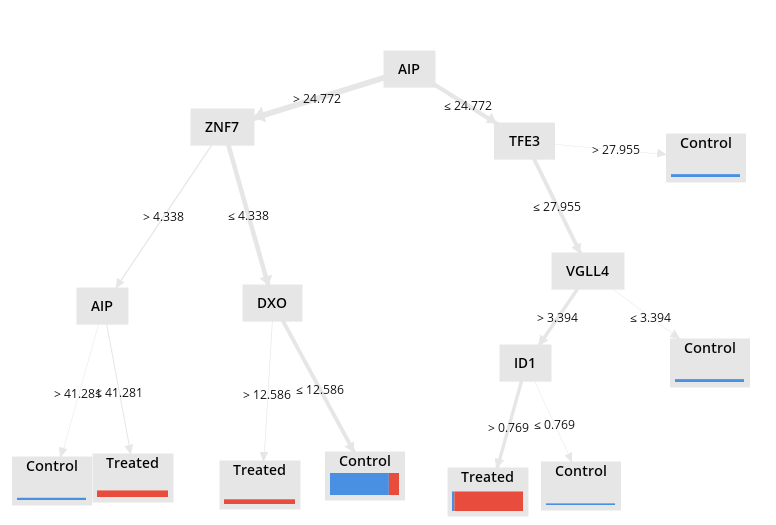

Supplement: Supplementary file 1 [file cells-10-03139-s001.zip › cells-1423536-supplementary/Supplementary File 1/RF_accuracy/RF_accuracy.png]

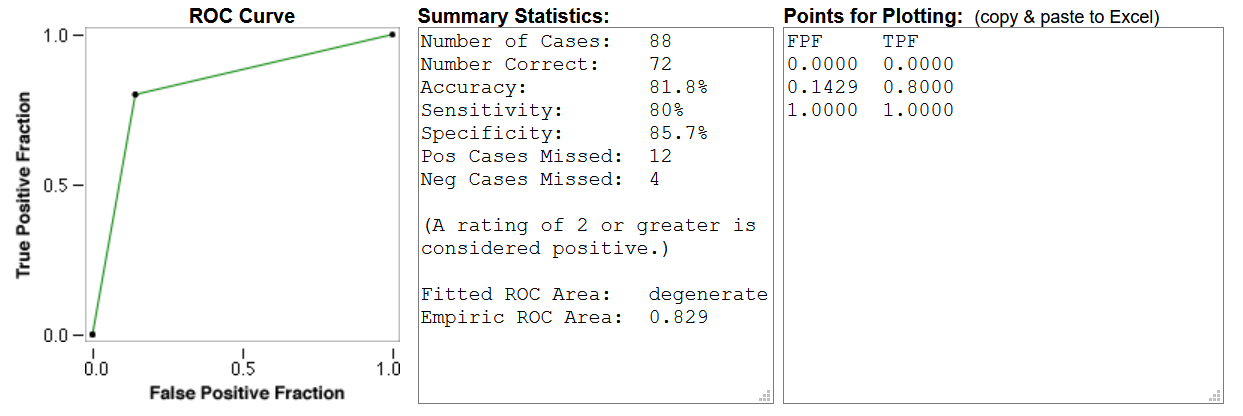

Supplement: Supplementary file 1 [file cells-10-03139-s001.zip › cells-1423536-supplementary/Supplementary File 1/RF_accuracy/ROC RF_accuracy.png]

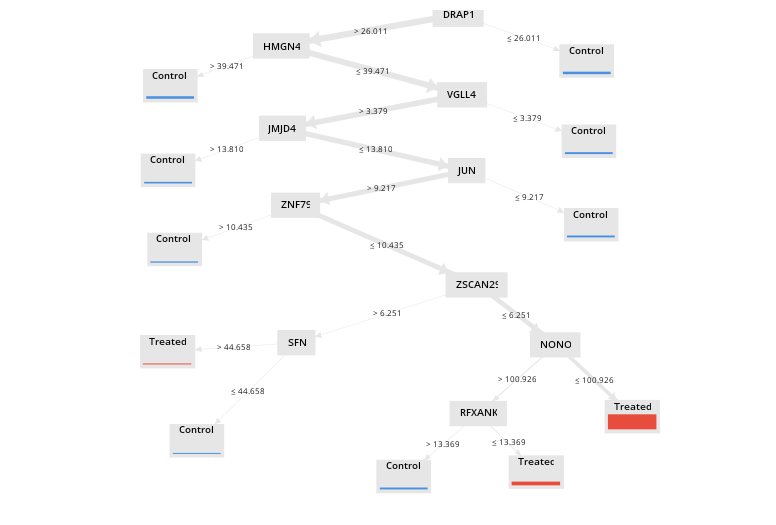

Supplement: Supplementary file 1 [file cells-10-03139-s001.zip › cells-1423536-supplementary/Supplementary File 1/RF_gain ratio/RF_gain ratio.png]

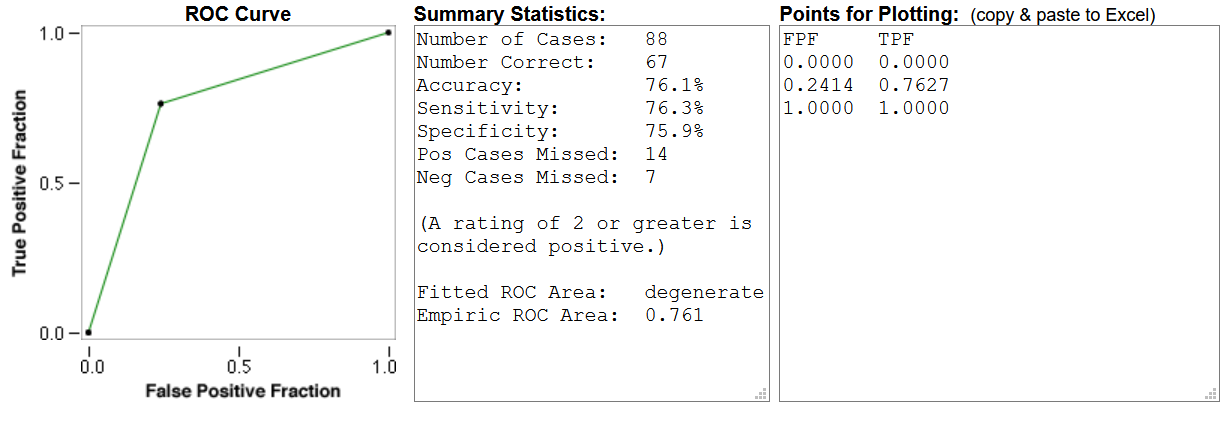

Supplement: Supplementary file 1 [file cells-10-03139-s001.zip › cells-1423536-supplementary/Supplementary File 1/RF_gain ratio/ROC RF_gain ratio.png]
